# ATP Reduces the Entry of Calcium Ions into the Nerve Ending by Blocking L-type Calcium Channels

**Published:** 2018

**Authors:** E. F. Khaziev, D. V. Samigullin, A. N. Tsentsevitsky, E. A. Bukharaeva, E. E. Nikolsky

**Affiliations:** Kazan Institute of Biochemistry and Biophysics, FRC Kazan Scientific Center of RAS, P.O. Box 30, Kazan, 420111, Russia; Institute of Fundamental Biology and Medicine, Kazan Federal University, Open Lab “Neuropharmacology”, Kremlevskaya Str. 18, Kazan 420008, Russia; National Research Technical University named after A.N. Tupolev, K. Marx Str. 31/7, Kazan 420111 , Russia

**Keywords:** ATP, calcium transient, calcium channels, neuromuscular junction

## Abstract

At neuromuscular junctions, ATP inhibits both the evoked and spontaneous acetylcholine release and inward calcium current operating via presynaptic P2Y receptors. It was shown in the experiments with the frog neuromuscular synapse using specific calcium-sensitive dye Oregon Green Bapta 1 that exogenous ATP reduces the amplitude of calcium transient, which reflects the changes in the entry of calcium ions in response to the nerve pulse. The depressing effect of ATP on the transient was prevented by suramin, the blocker of P2 receptors. Nitrendipine, a specific blocker of L-type calcium channels, *per se *decreased the calcium transient amplitude and significantly attenuated the effect of ATP on the calcium signal. Contrariwise, the preliminary application of ATP to the neuromuscular junction completely eliminated the depressing effect of nitrendipine on the calcium response. The obtained data suggest that an essential component in the inhibitory action of ATP on the calcium transient amplitude is provided by reduction of the entry of calcium ions into a frog nerve ending via L-type voltage-gated calcium channels.

## INTRODUCTION

ATP reduces the amplitude of multiquantal endplate currents (EPCs) in the neuromuscular junction by activating presynaptic P2Y receptors [1]. The inhibitory activity of ATP on the amplitude of postsynaptic currents is a presynaptic effect and can be caused by changes in the activity of calcium channels, the input of calcium (Ca^2+^) through which exocytosis of synaptic vesicles is triggered. Indeed, ATP reversibly reduces the Ca^2+^ current in the perisynaptic region of axon [2] and decreases the amplitude of Ca^2+^ transient recorded in various regions of a frog nerve terminal [3]. A change in the transient amplitude reflects changes in the concentration of free Ca^2+^ ions in the terminal [4, 5], while its decrease in association with ATP action may be indicative of the effect of this purine on the activity of presynaptic calcium channels. There are several types of voltage-gated calcium channels that function in a frog nerve terminal [4]. It remains unknown the activity of which type of channels is altered by ATP. The data regarding the effect of ATP on L-type voltage-gated calcium channels are quite contradictory. It has been shown on various objects that ATP is capable of both enhancing the entry of calcium ions through L-type channels [6] and inhibiting these channels [7]. In this study, we used the fluorescent method of recording Ca^2+^ transient in a frog nerve terminal to find whether or not presynaptic L-type voltage-gated calcium channels are involved in ATP-induced reduction of the Ca^2+^ transient amplitude. It was established that the decrease in the transient amplitude upon activation of purine receptors is partially due to a reduction in the entry of Ca^2+^ ions through L-type calcium channels.

## EXPERIMENTAL SECTION

The study was performed using an isolated neuromuscular specimen of the *m. cutaneus pectoris *obtained from the frog *Rana ridibunda*. The relative change in the Ca^2+^ level in the nerve terminal (Ca^2+^ transient) was evaluated using the Oregon Green Bapta 1 fluorescent dye. The technique of dye loading through the nerve stump and the protocols of recording and processing fluorescent signals were described in detail in [8]. The experimental protocol was as follows. After the fluorescent dye was loaded into the nerve terminals, the specimen was placed in a 3-ml reservoir through which a perfusion solution was fed at a rate of 3 ml/min. In order to prevent contractions of muscle fibers upon motor nerve stimulation, a Ringer’s solution with a reduced content of Ca^2+^ ions and increased concentration of Mg^2+^ ions (113.0 mM NaCl, 2.5 mM KCl, 3.0 mM NaHCO_3_, 6.0 mM MgCl_2_, 0.9 mM CaCl_2_; pH 7.2–7.3; temperature, 20.0 ± 0.3°C) was used. The experiments were conducted in accordance with the ethical principles and guidelines recommended by the European Science Foundation (ESF) and the Declaration on Animal Welfare. A total of 6–21 synapses obtained from 3–5 animals were used in each series of experiments.

Stimulation of the motor nerve with rectangular pulses 0.2 ms long, with a frequency of 0.5 imp/s, was performed by a stimulator (A-M Systems 2100) using a suction electrode. A total of 60 consecutive fluorescent signals were recorded along the entire length of the selected nerve terminal under control conditions; the test substance was then added to the perfusion solution. Registration of 60 signals from the same terminal as the one used in the control was initiated 20–25 min after substance application. If necessary, the next test substance was added to the perfusion solution in the presence of the first substance, and all signals from the same nerve terminal were recorded again 20–25 min later. Preliminary experiments were conducted; the results indicated that the amplitude-time parameters of the fluorescent signal in response to infrequent stimulation of the motor nerve do not undergo any changes for a period of 3–4 h.

Fluorescent signals in response to a nerve stimulus were recorded using a photometric unit based on an Olympus BX-51 microscope with a 60× water immersion lens and the Turbo-SM software. A Polychrome V monochromator (Till Photonics, Munich, Germany), tuned to the excitation wavelength of the dye, 488 nm, was used as a source of illumination. The fluorescent signal was isolated using the following set of filters: 505DCXT dichroic mirror, E520LP emission (Chroma). The area of illumination was restricted by a diaphragm in order to reduce background illumination. The data were analyzed using a Neuro CCD camera and the ImageJ software. The terminal and background areas were defined using the ImageJ software. Background fluorescence was subtracted from all the fluorescence values of the terminal area. The data are represented as a (Δ*F */ *F*_0_ – 1) × 100% ratio, where Δ*F *is the fluorescence intensity in response to the stimulus and *F*_0_ is the fluorescence intensity at rest. *F*_0_ was registered before each recording of fluorescent signals in response to a nerve stimulus.

The statistical significance of the differences between the samples was estimated using the Student’s t-test and the Mann–Whitney U test. Differences between the samples were considered statistically significant at *p *= 0.05 (where *n *is the number of studied synapses).

## RESULTS AND DISCUSSION

**Fig. 1:**
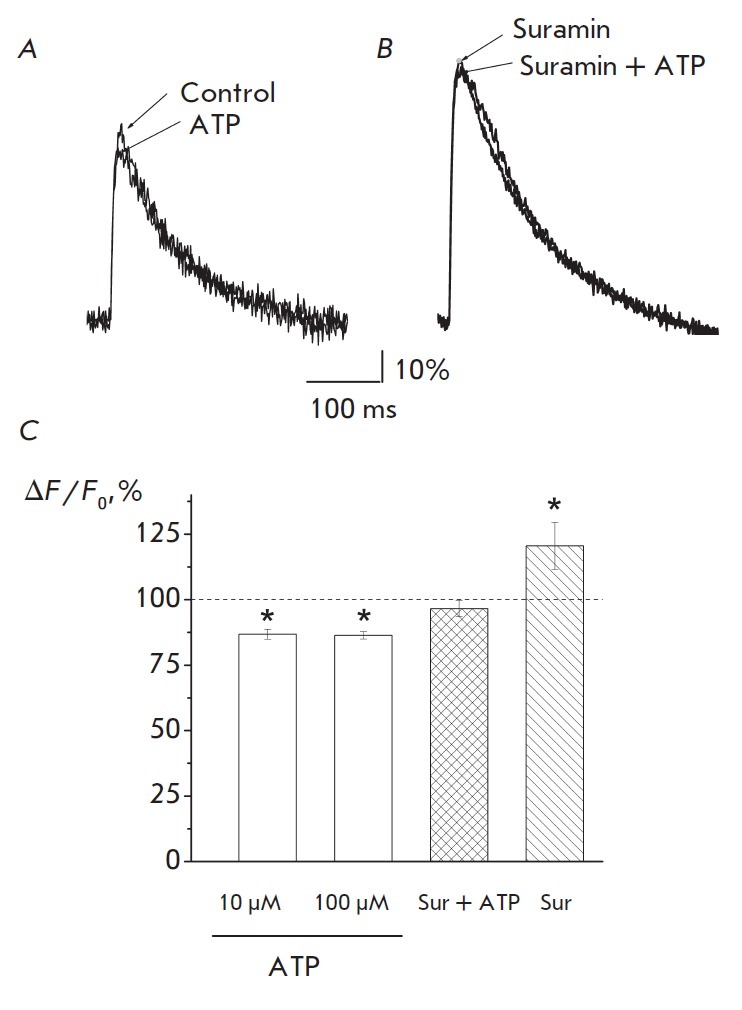
ATP reduces the amplitude of calcium transient operating via P2 receptors. *A *– The effect of 10 μM ATP on Ca^2+^ transient. *B *– The absence of ATP effect on transient in the presence of suramin. *A, B *– the averaging of 60 fluorescence signals. C – Effect of ATP (10 and 100 μM), suramin (Sur), and ATP in the presence of suramin on the amplitude of Ca^2+^ transient. The averaged changes in the Ca^2+^ transient amplitude for ATP and suramin (Sur) are expressed as a percentage of related calcium signal amplitudes under control conditions. In the case of combined action of suramin and ATP (Sur+ATP), the amplitude of Ca^2+^ transient under suramin was taken as 100%. * *p * < 0.05

Exogenous ATP at a concentration of 10 μM decreased the amplitude of Ca^2+^ transient in response to a nerve stimulus by an average of 13.2 ± 1.9% (*p *= 0.0003, *n *= 8; *Fig. 1 A,C*). An increase in ATP concentration to 100 μM did not affect the intensity of this effect: the Ca^2+^ transient was decreased by 13.6 ± 1.4% (*p *= 0.000003, *n *= 21) relative to the reference values (*Fig. 1C*).

Suramin, a non-selective P2 receptor antagonist, at a dose of 300 μM increased the Ca^2+^ transient value by an average of 20.5 ± 9.0% (*p *= 0.037, *n *= 8; *Fig. 1C*) relative to the control values. Addition of 100 μM ATP to the medium containing suramin did not significantly alter the Ca^2+^ transient, which was equal only to 103.4 ± 3.1% (*p *= 0.27, *n *= 5; *Fig. 1 B,C*). Thus, the effect of ATP on the amplitude of Ca^2+^ transient is associated with the activation of P2 receptors.

**Fig. 2:**
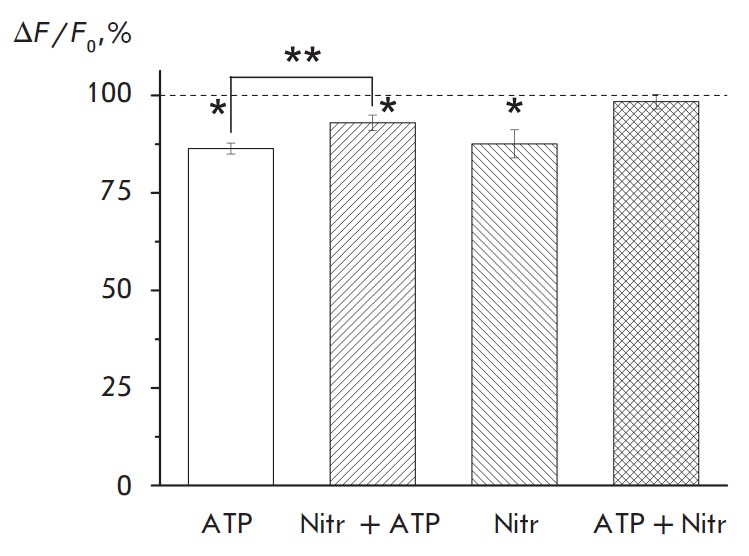
ATP-induced reduction in the calcium transient amplitude is associated with blockade of presynaptic L-type voltage-gated calcium channels. The depressing effect of ATP is attenuated after the preliminary blocking of L-type calcium channels by nitrendipine (Nitr+ATP). Nitrendipine *per se *decreases the amplitude of Ca^2+^ transient (Nitr). Nitrendipine does not alter the amplitude of transient under conditions when P2 receptors are activated by ATP (ATP+Nitr). * *p * < 0.05

A specific L-type calcium channel blocker, nitrendipine, at a concentration of 5 μM reduced the amplitude of Ca^2+^ transient by 12.4 ± 3.6% (*p *= 0.0003, *n *= 12; *Fig. 2*), indicative of the contribution of L-type channels to the overall calcium current caused by the action potential (see also [4]). The change in the Ca^2+^ transient amplitude caused by ATP under L-type calcium channel blockade was only 4.2 ± 1.1% (*p *= 0.016, *n *= 7; *Fig. 2*), which is much less than the effect of intrinsic ATP (Mann–Whitney U test, *p *= 0.011). Thus, the blockade of L-type calcium channels alleviated the decrease in the Ca^2+^ transient amplitude through ATP action. One can assume that activation of purine receptors by ATP leads to the suppression of L-type calcium channels. If this assumption is true, then a decrease in the transient amplitude induced by nitrendipine should be less pronounced in the presence of ATP. Indeed, nitrendipine did not affect the Ca^2+^ transient amplitude after preliminary ATP application: the amplitude only changed by 2.0 ± 1.9% (*n* = 6; *Fig. 2*).

We have showed earlier that exogenous ATP at a concentration of 100 μM reduces the Ca^2+^ transient amplitude equally in different regions of the extended nerve terminal of a frog [3]. The reduction in the transient caused by ATP corresponds to a decrease in the amplitude of induced EPC at normal calcium content [1] and the quantal content of EPC at reduced Ca^2+^ ion concentration in solution [9]. The data on the ATP-induced decrease in the input of Ca^2+^ ions into the terminal in response to a nerve impulse are consistent with the results reported in [2], where ATP was shown to cause a reversible decrease in the presynaptic calcium current.

Exogenous ATP reduced the amplitude of EPC as a result of the activation of presynaptic P2Y receptors, since the effect was prevented by preliminary incubation of the specimen in suramin [1]. In our experiments, the ATP-induced decrease in the Ca^2+^ transient amplitude was also prevented by suramin (*Fig. 1 B,C*). At the same time, suramin *per se *enhanced Ca^2+^ transient (*Fig. 1*). This effect can be associated with the possibility of increasing the concentration of Ca^2+^ ions in the cytoplasm as a result of its release from the sarcoplasmic reticulum [10]. It is shown that suramin increases not only the probability of an open state, but conductance of single ryanodine-sensitive channels as well [11]. The suramin-induced elevation of the Ca^2+^ transient amplitude is consistent with the data on the increase in the quantal content of EPC observed upon blockade of P2 receptors [12].

The data on the effect of ATP on L-type voltage-gated calcium channels are quite contradictory. In micromolar concentrations, ATP is capable of suppressing the current through the L-type Ca^2+^ channels in cardiomyocytes in a reversible, dose-dependent manner [7]. Meanwhile, activation of purine receptors can enhance the entry of Ca^2+^ ions through the L-type channels in the perisynaptic glial cells of a frog [6]. Our results demonstrate that ATP-induced decrease in the Ca^2+^ transient amplitude is influenced by a suppression of the L-type Ca^2+^ channel activity. This is evidenced by a significant decrease in the effect of ATP on Ca^2+^ transient in the presence of nitrendipine, a specific blocker of L-type channels (*Fig. 2*). An additional confirmation to the fact that L-type Ca^2+^ channels contribute to the ATP action is that their specific blocker, nitrendipine, does not affect the transient amplitude after the pre liminary action of ATP (*Fig. 2*). Under these conditions, when the activity of L-type Ca^2+^ channels has been already reduced by ATP, nitrendipine does not have any target for action.

Our results demonstrate that the activity of L-type voltage-gated calcium channels plays a crucial role in the inhibitory effect of ATP on the Ca^2+^ entry into the nerve terminal of a frog in response to a nerve stimulus.
